# Circulatory microRNA expression profile for coronary artery calcification in chronic kidney disease patients

**DOI:** 10.4314/ahs.v21i2.31

**Published:** 2021-06

**Authors:** Bhooma Vijayaraghavan, Sridharan Jeyamohan, Giri Padmanabhan, Antony Joseph Velangann, Kumaresan Ramanathan

**Affiliations:** 1 Kidney care, 10th B cross, Thillai Nagar, Tiruchirappalli-620018, Tamilnadu, India; 2 Department of Biochemistry, Bharathidasan University, Tiruchirappalli-620024, Tamilnadu, India; 3 Department of Medical Biochemistry, School of Medicine, College of Health Sciences, Mekelle University (Ayder Campus), Mekelle, Ethiopia

**Keywords:** CKD, CAD, microRNA, coronary artery calcification

## Abstract

**Background & Aim:**

Coronary artery disease (CAD) is the primary cause of mortality in patients with end stage renal disease (ESRD). MicroRNA profiling is proven as a powerful tool in the diagnosis of any disease at the molecular level. Hence, the present study aimed to profile the microRNA expression for CAD especially coronary artery calcification in CKD patients.

**Materials and Methods:**

Two hundread patients with CKD stages 3 to 5 without dialysis and healthy controls were included in this study. All two hundred patients underwent 1024 multi sliceardiac computed tomography (CT) scan for calcium scoring. The calcium scoring more than 100 have been included in the study. We performed miRNA microarray analysis from serum samples of seven high calcium scored with CKD patients and one control patients.

**Results:**

Seven patients have observed circulating miRNAs has significantly upregulated and downregulated when compared with control patients. mir21, mir 67, mir 390, mir 56, mir 250, mir 65 and mir 13 were up regulated and mir235, mir256, mir226, mir207, mir255, mir193 were downregulated. There was no significant difference in left ventricle function.

**Conclusion:**

13 microRNAs play a potential role in coronary artery calcification in CKD patients.

## Introduction

Chronic kidney disease is a major global health problem^1^ and it is an independent risk factor for the development of coronary artery disease (CAD).^2^ Coronary artery disease is the leading cause of morbidity and mortality in patients with CKD.^3^ Data from prospective studies support that cardiovascular diseases (CVD) remain the most common cause of morbidity and mortality in patients with CKD and end-stage renal disease (ESRD).^4^ The spectrum of CVD not only involves obstructive coronary artery disease (CAD), but also involves other disease states such as chronic heart failure, sudden death, and arrhythmias. The main factors for the sensitive risk in this population, beside advanced age and a high proportion of diabetes and hypertension, are malnutrition, chronic inflammation, accelerated atherosclerosis, highly prevalent endothelial dysfunction (ED), coronary artery calcification (CAC), left-ventricular structural and functional abnormalities and bone mineral disorders (BMD).^5^–^7^ Coronary artery calcium (CAC) score is an independent predictor of cardiac events in both the general population and CKD patients.^1^ The prevalence of CAC in different stages of CKD varies from 13.9% in stages I and II, up to 83% in stages III-V. The prevalence and extent of CAC are increased in patients with ESRD even in young adults.^8^ The Dallas Heart Study showed that CAC scores >400 were 8-fold more prevalent in stages III-V CKD compared with patients without CKD. This association is substantially stronger in diabetics.^9^ The calcification could be reduced by using magnesium chloride and sodium thiosulfate. However, there are no pharmaceutical drugs available to reduce calcium deposits. Experiments using calcium channel blockers or statins have not been convincing.^10^,^11^ Some current medical treatments, however, may be associated with an increased risk of calcification. For example, treatment of patients for recurrent thrombosis with coumarins results in accelerated calcification.^12^,^13^ Similarly, in end stage renal disease, treatment of hyperphosphatemia with phosphate binders that contain calcium have been associated with more CAC compared to treatments with non-calcium-based phosphate binders.^14^ In patient groups with a strongly elevated risk for arterial calcification, patient-specific measures could possibly prevent arterial calcifications. CAD is hard to diagnose without the help of the well-established invasive coronary angiogram (CAG) technique. Although ECG and ETT have been widely used, there is no specific plasma biomarker for the clinical diagnosis of CAD especially calcium deposition on arteries, particularly for the early diagnosis of CAD. Therefore, there is a clinical demand for specific and reliable non-invasive, innovative, molecular biomarkers for the early diagnosis of CAD specifically calcification on arteries as well as therapeutic targets.

Circulating microRNAs (miRNAs) have attracted major interest as novel biomarkers for the early diagnosis of CAD.^15^ miRNAs are a class of small (B22 nucleotides long), highly specific, endogenous, single-stranded, non-coding RNAs that regulate the expression of target genes by binding to the 3′ untranslated region and degrading or inhibiting the translation of mRNAs.^16^ It is well established that miRNAs play critical roles in physiological and pathological processes in the cardiovascular system, such as endothelial dysfunction, inflammation, apoptosis, angiogenesis, atherosclerosis, and neointimal hyperplasia or restenosis.^17^–^20^ However, there are no reports regarding circulating miRNAs as non-invasive biomarkers for the diagnosis of coronary artery calcification in CKD patients.

The purpose of the present study was to use human CKD serum samples to profile the circulatory microRNA expression which have a putative involvement in calcification and provide new platform for these microRNAs may serve as targets for subsequent diagnostic and recognised therapeutic studies in CKD patients.

## Materials and methods

### Study population and design

Two hundred patients with CKD stages 3 to 5 age ranged from 48 years to 65 years and healthy control were included in this case control observational study from Dr.Giri's out patient clinic (OPD), Tiruchirappalli, India during January 2018 to December 2018. The study participants who were age >30 years, calcium score >100, willing to provide written and informed consent were included and age >65 years, history or clinical features of cardiac failure, previous heart surgeries, active malignancies and non willing participants were excluded from the study.

### Calcium scoring

All two hundred patients underwent multi slice cardiac computed tomography (CT) scan for calcium scoring and it was done by the same radiologist. Administration of 20 mg metoprolol if patients had a heart rate more than 70 beats/min prior to the scan and administration of sublingual nitroglycerin 0.8 mg for all patients. Performed a scan without contrast dye to calculate total calcium score (Iwasaki K et al., 2011) and expressed as Agatston score (Agatston AS et al.,1990). The participants whose coronary calcium scoring ≥100 has been at very high cardio-vascular risk (Valensi P et al., 2018).

10 ml of blood samples were drawn from patients those who have calcium score more than 100 and centrifuged for serum according to a standardized protocol. This study was approved by the hospital ethics committee and written informed consent has been obtained from all the study population prior to the study. Also, the study was performed according to the principles laid out in the Declaration of Helsinki. Similarly, patients samples were analysed the routine parameters like haemoglobin, blood sugar, serum urea, creatinine, eGFR, Total protein, albumin, globulin, calcium, phosphorous, Ca*PO4, Alakline phosphatase, uric acid, electrolytes and 2D echo cardiography has been done all the study participants by a same cardiologist for assessment of ejection fraction.

### miRNA Microarray profiling Using Agilent Platform

#### Microarray: Labelling and hybridization

The miRNA labelling was performed using miRNA Complete Labelling and Hyb Kit (Agilent Technologies, Part Number: 5190-0456). The total RNA sample was diluted to 100ng/ul in nuclease free water. About 200ng of total RNA was dephosphorylated using Calf Intestinal Alkaline Phosphatase (CIP) master mix (Agilent Technologies, Part Number: 5190-0456) by incubating at 37°C for 30 minutes. The dephosphorylated miRNA sample was denatured by adding Dimethyl Sulfoxide and heating at 100°C for 10 minutes and transferred to ice-water bath. The Ligation master mix (Agilent Technologies, Part Number: 5190-0456) containing Cyanine 3-pCp was added to the denatured miRNA sample and incubated at 16°C for 2 hours. The Cyanine 3-pCp labelled miRNA sample was dried completely in the vacuum concentrator (Eppendorf, Concentrator Plus, Catlog Number 5305000) at 45°C for 2 hour. The dried sample was suspended in nuclease free water and mixed with Hybridization Mix containing blocking solution (Agilent Technologies, Part Number: 5190-0456) and Hi-RPM Hybridization Buffer (Agilent Technologies, Part Number: 5190-0456) and incubated at 100°C for 5 minutes followed by snap chill on ice for 5 minutes. The samples were hybridized on the Human miRNA 8x60K Arrays. The hybridization was carried out at 55°C for 20 hours. After hybridization, the slides were washed using Gene Expression Wash Buffer1 (Agilent Technologies, Part Number 5188-5325) at room temperature for 5 minutes and Gene Expression Wash Buffer 2 (Agilent Technologies, Part Number 5188-5326) at 37oC for 5 minutes. The microarray slide was scanned on a G2600D scanner (Agilent Technologies)

### Microarray Data Analysis

Data extraction from Images was done using Feature Extraction software v 11.5.1.1 of Agilent. Feature extracted raw data was analyzed using Gene Spring GX Version 12.0 software from Agilent. Normalization of the data was done in Gene Spring GX using the 90th percentile shift (Percentile shift normalization is a global normalization, where the locations of all the spot intensities in an array are adjusted. This normalization takes each column in an experiment independently and computes the ηth percentile of the expression values for this array, across all spots (where n has a range from 0–100 and n=90 is the median). It subtracts this value from the expression value of each entity) and normalized to Specific control Samples. Significant miRNA up and down regulated in test samples with respect to control sample were identified. Statistical T-test p-value was calculated based on volcano Plot. The gene targets for the differentially regulated miRNA were identified using Target Scan database http://www.targetscan.org/ which is integrated in Gene Spring GX software. The differentially expressed miRNA were clustered using hierarchical clustering based on Pearson coefficient correlation algorithm to identify significant miRNA expression patterns across the different conditions of the experiment.

## Results

There were no significant correlation with ejection fraction and other parameters. The baseline characteristics of the study participants were shown in the table 1.

**Table 1:** Patient's baseline characteristics

Parameters	Patient Group (N:07)
Age years	56.57±6.02
**Calcium score**	**843 ±190.23**
**EF %**	**54.66 ± 21.93**
**HB gms%**	**7.35 ± 1.58**
Sugar mg/dl	202.2 ±104.85
Urea mg/dl	160.33 ±112.75
**Creatinine mg/dl**	**4.97 ±2.87**
**eGFR ml/min**	**26.71 ± 14.64**
Total Protein gms%	5.53 ±0.70
Albumin gms%	2.43 ±0.76
Globulin gms%	3.1 ± 0.55
Calcium mg/dl	7.8 ±1.32
Phosphorous mg/dl	5.77 ± 1.24
Ca*PO4	44.62 ±10.12
Alkaline Phosphatase U/L	167.25 ±96.93
Uric Acid mg/dl	6.67 ± 3.50
Na mEq/L	136.5 ±8.96
K mEq/L	4.55 ± 1.04
HCO3 mmol/L	11.66 ± 2.88
Cl mmol/L	99 ±14.52

The microarray cohort of subjects included 8 individuals and their Samples were labelled using the Agilent's Quick-Amp labelling Kit. Quality control was performed using Nanodrop (Table 2). The results were submitted in NCBI and obtained miRNA gene expression omnibus number (GSE89699) successfully.

**Table 2:** Nanodrop Analysis of labelled cRNA

Sample ID	Dye	RNA Concentration ng/µl	Absorbance value 260/280	Absorbance value 260/230	Total yield Ng
1A	pCp-Cy3	32.00	1.39	0.33	480
2A	pCp-Cy3	18.30	1.63	0.06	274.5
3A	pCp-Cy3	46.80	1.50	0.57	702
4A	pCp-Cy3	14.60	1.26	0.34	365
5A	pCp-Cy3	89.10	1.44	0.52	1336.5
6A	pCp-Cy3	9.90	1.32	0.45	247.5
7A	pCp-Cy3	68.30	1.75	0.71	1024.5
9A Dnase treated	pCp-Cy3	10.1	1.70	0.2	252.5

### Expression profiles of microRNAs in the plasma of individuals with CAC

**Fig. 1:**
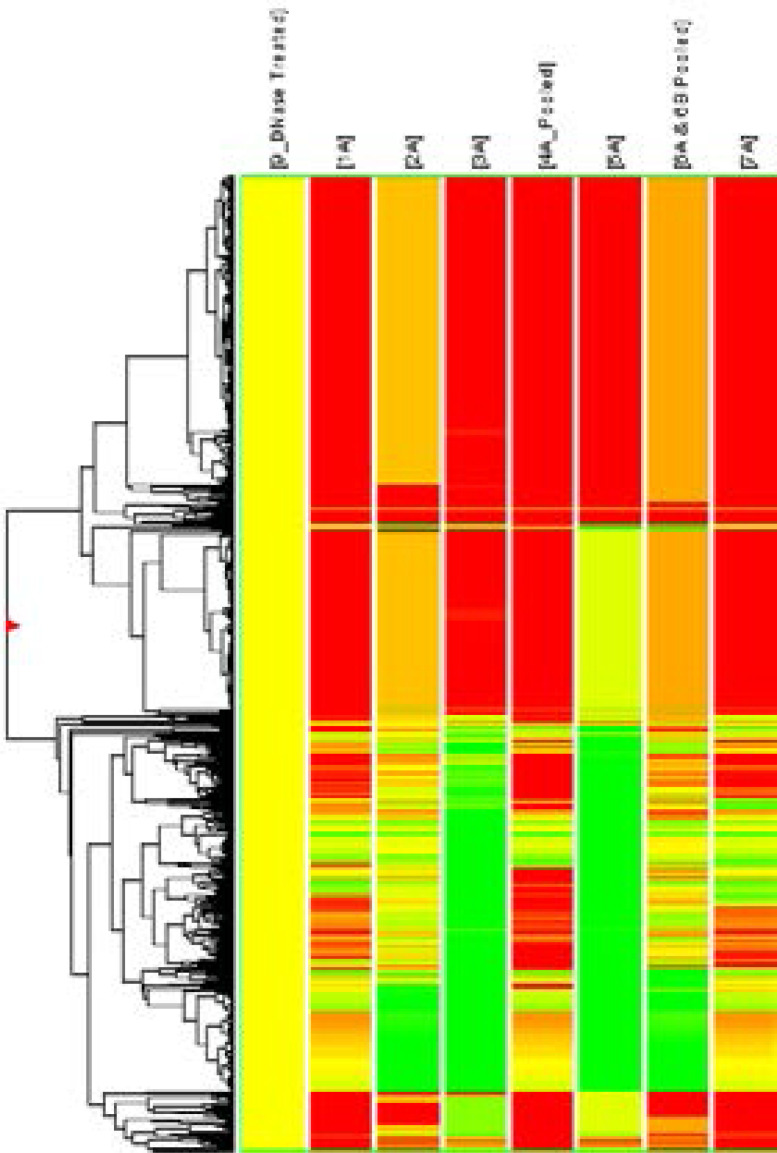
Heat map of microRNA (miRNA) microarray expression data from blood samples of Individuals with (n = 7) and without (n = 1) coronary artery calcification. Hierarchical clustering of miRNA is based on similar expression profiles in test vs. Control. Clustering analysis was performed using GeneSpringGX Software using Average Linkage rule with Pearson centered Distance Metric. Red color in the cluster indicates up regulation in test and green color in the cluster indicate down regulation in test compare to control. The Fold expression values represented in the cluster are in terms of log base 2.

To evaluate the differential miRNA levels in individuals with CAC, we comparatively profiled plasma miRNA expression of 7 and 1 individuals with and without CAC, respectively. The levels of circulating miRNAs significantly upregulated and downregulated among CAC patients, as illustrated in the heat map shown in Fig. 1. Levels of 7 miRNAs were upregulated and those of 6 miRNAs were down regulated in the CAC patients (Table 3). After evaluating the differential miRNA expression pattern, it showed significant positive correlation with CACS.

**Table 3:** Differentially regulated miRNA

Samples	Up Regulated	Down Regulated
1A	21	255
2A	67	236
3A	390	226
4A	56	207
5A	250	255
6A	65	193
7A	13	256

## Discussion

MiRNAs have been demonstrated to play crucial roles in many physiological and pathophysiological processes.^21^ miRNAs contribute to different forms of CVD, and the changes in circulating miRNAs can be detected because of pathological changes.^22^,^23^ Circulating miRNAs have been identified as biomarkers for various physiological and pathological conditions.^22^,^24^ The microarray chip for miRNA provides a powerful approach for global circulating miRNA characterization, and it is simple to universally perform quantitative validation using real time-PCR.23 It has been suggested that the discovery-validation procedure for circulating miRNA biomarkers will be more efficient than that for traditional proteomic biomarker identification. To best of our knowledge, the function of miR-67, miR-390, miR-56, miR-250, miR-65, miR-13, miR-226, miR-236, miR-207, miR-256 remains unknown. Functional analysis in target Scan 6.0 showed that miR-21 and miR-255 could target different genes involved in increased the Wnt pathway in hepatocellular carcinoma. miR-193 involved in cause of primary segmental glomerulosclerosis.

The study provided significant clinical significance, to our knowledge, this is the first study to evaluate the circulating miRNA profile of CAC in patients with CKD. Calcification of the coronary arteries is highly correlated with atherosclerosis.^25^,^26^ Early detection of CAC is important for identifying subclinical atherosclerosis and predict the risk of CAD.^25^,^27^ By identifying specific circulating miRNAs for CAC, we are providing a novel way of identifying the severity of CAC which can also function as potential biomarkers for the presence of obstructive CAD. Moreover, the results of the current study highlight that further insight into the function of circulating miRNAs in the process and progression of CAC.

## Conclusion

All these 13 micro RNAs are play a key role in coronary artery calcification in patients with CKD. However, larger population studies are required to confirm our results.
